# An acceptance analysis of a sexual health education digital tool in resource-poor regions of Kenya: an UTAUT based survey study

**DOI:** 10.1186/s12905-023-02839-6

**Published:** 2023-12-19

**Authors:** Clarissa Soehnchen, Vera Weirauch, Rebecca Schmook, Maike Henningsen, Sven Meister

**Affiliations:** 1 Faculty of Health Informatics / School of Medicine, Witten/Herdecke University, Witten/Herdecke, Deutschland; 2 Department Health Care, Fraunhofer Institute for Software and Systems Engineering ISST, Dortmund, Germany

**Keywords:** Sexual health information, Africa, Young women, UTAUT, SEM, Multiple regression analysis, Confirmatory factor analysis, SUS, mHealth, Digital health intervention, Kenya, Digital health tools, Sexual health education

## Abstract

**Background:**

Unplanned pregnancies and sexually transmitted diseases are a concern in Sub-Saharan Africa, particularly in low-income areas. Access to sexual health information is limited, partly due to the absence of comprehensive sex education in the national school curriculum and social taboos. In response to these challenges, this study introduces a web-based prototype, designed to provide essential sexual health information, targeting 18 to 35-year-old Kenyans, focusing on contraception, menstruation, and female genital mutilation.

**Method:**

Aiming to investigate young adults’ behavioral intention to use a digital tool for sexuality education, by analyzing factors affecting acceptance and usability in low-income and resource-poor regions in Kenya. To explore the acceptability and use of the developed digital tool, this study used a modified version of the Unified Theory of Acceptance and Use of Technology (UTAUT), complemented by the System Usability Scale (SUS) questionnaire. For statistical analysis, a Structural Equation Model (SEM) including Confirmatory Factor Analysis (CFA) and Linear Regression was used. Regarding the reporting of the E-survey results, the Checklist for Reporting Results of Internet E-surveys (CHERRIES), was considered.

**Results:**

Survey information from 77 persons (69 female, 7 male, 1 diverse) were collected. A modified UTAUT appears as an appropriate model for measuring the constructs and integrating evidence-based approaches to advanced and safe sexual healthcare information. Results from the SEM showed perceived usefulness, attitude towards healthcare integrated evidence technology, and usability as well as having a significant positive impact on the acceptance, the intention to use as well as wellbeing. Having the resources and knowledge necessary for the usage of a digital tool turns out to have a significant negative impact. A SUS score of 67.3 indicates the usability of the tool for sexual health information, assessed as okay.

**Conclusions:**

The study adopts validated methods to assess the acceptability and usability of a digital sexual health education tool in Kenya. Emphasizing its potential effectiveness and highlighting the influence of cultural and contextual factors on technology adoption.

## Background

Unplanned pregnancies and the prevalence of sexually transmitted diseases are significant concerns in Sub-Saharan Africa, where approximately half of all pregnancies among young women in low-income areas occur without planning [1–3]. Moreover, two-thirds of new cases of sexually transmitted infections originate from this region [4], and over 80% of individuals report experiencing technology-facilitated sexual violence, gender-based violence, or other forms of violence [4, 5]. Given the challenging circumstances in Sub-Saharan Africa, it is imperative to reduce the incidence of unplanned pregnancies and the spread of diseases. This involves ensuring that adolescents have access to information about contraception [5] and learn about sexual health to be able to make educated choices.

Access to knowledge empowers individuals to make informed and sustainable decisions regarding their bodies and health. Unfortunately, adolescent, and especially Kenyan women, often lack access to essential healthcare information [6]. The lack of access to structured, verified, valid, and reliable sexual information, especially concerning contraception, menstruation, and female genital mutilation, leaves young women vulnerable to health risks [7]. This issue is multifaceted. Reasons being, the official national school curriculum in Kenya does not include comprehensive sex education, resulting in teachers being ill-prepared to address these topics in the classroom [8]. Furthermore, sexual health matters are influenced by religious, tribal, and social affiliations, which can lead to varied or suppressed discussions [7, 9]. The societal taboo surrounding contraceptive practices can even lead to fatal abortions to prevent unwanted pregnancies [10]. This primarily affects young Kenyan women between the ages of 18–35 in resource-poor rural areas, creating a vicious cycle.

Considering the challenges in lack of infrastructure and limited financial resources and access to education and sexual health information, where on the contrary the increased use of the internet and digital technologies presents an opportunity for behavioral change [11]. Literature shows a growing focus on digital tools to address these problems [11, 10, 7, 12–16].

**Fig. 1:**
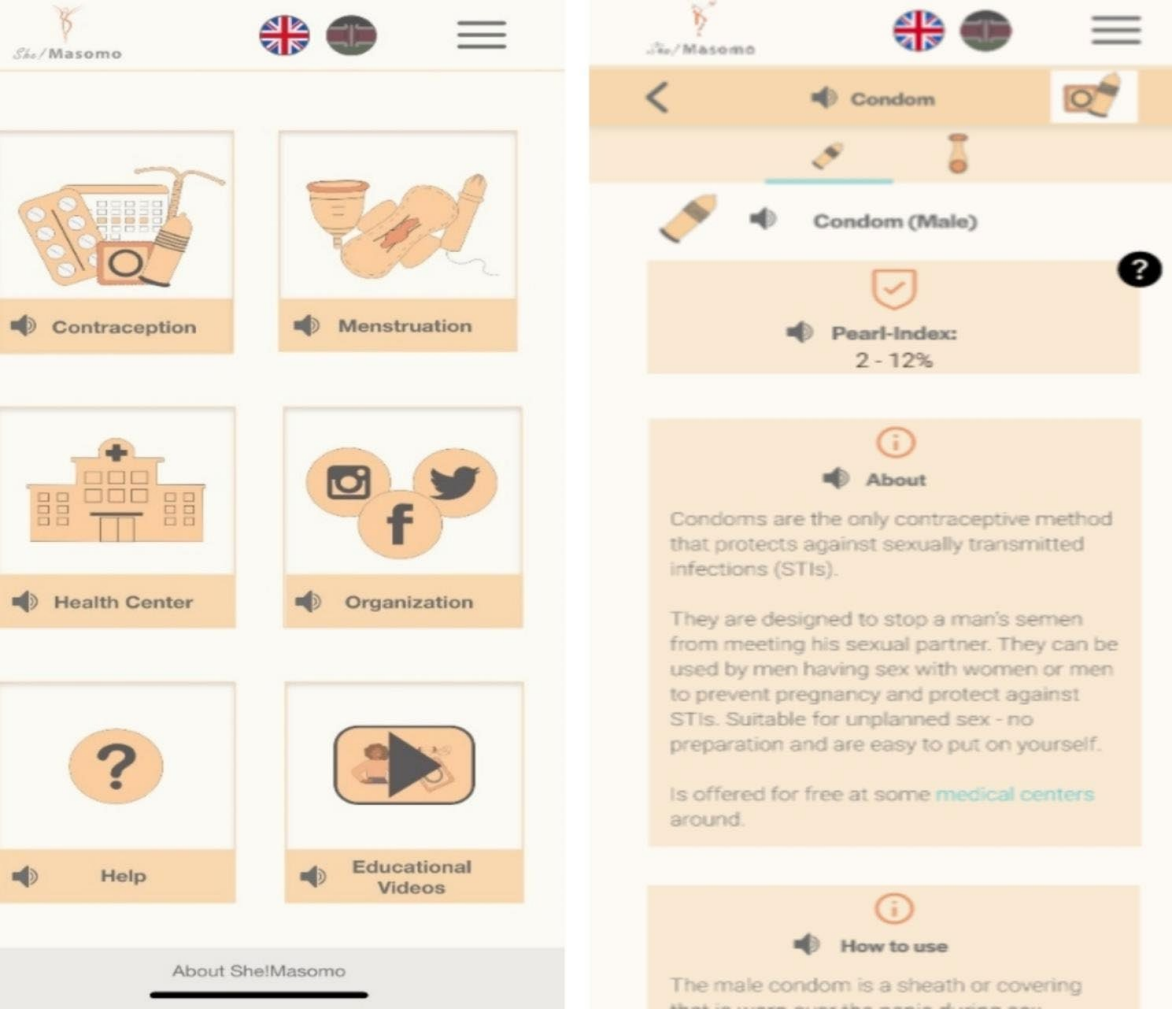
Prototype screenshot

The use of digital tools and social media has boosted confidence in this regard of allowing feedback and dispelling misconceptions [7, 11, 12]. Providing information can contribute to reducing the risks of sexually transmitted infections like HIV and unplanned pregnancies, with great potential for educating low-income and vulnerable communities [13, 14]. Despite a potential accessibility of digital sexual health educative tools, and willingness to share and engage with sensitive information, lasting user engagement is crucial [13]. Therefore, it is essential to explore innovative approaches to provide easily accessible knowledge for everyone via a digital tool in regarding sensitive information. The newly developed prototype, development in alignment with the double diamond established framework according to Soehnchen et al. [17]. serves for on-site te testing with a predefined target group in Kenya. The tool aims to be a secure platform for sensitive and intimate information. The prototype appears in form of a web-based application focusing on sexual health educative, contraception, menstrual period, and female genital mutilation information, available in English and Swahili (Fig. 1) with audio files. The tool is designed to address limited and stigmatized knowledge, among 18–35-year-old. Recognizing that the target audience has a different cultural background, and the design process originates from a European cultural perspective, it is crucial to involving potential end-users in the process. User involvement is a prerequisite for high acceptance, sustainable development, and successful implementation [14].

Therefore, the study employs a multimethod approach, combining a product-centered evaluation (usability test) with a user-centered evaluation (acceptance survey) to explore the acceptability and intention to use the developed digital tool.

### Objectives

A product-centered evaluation (usability test) and a user-centered evaluation (acceptance survey) took place, onsite in volunteer cooperation with local community centers in rural regions in Kenya. Therefore, the study aims to investigate [1] the acceptance of the given digital tool and the behavioral intention to use the tool for sexual health information of young women in Kenya and [2] investigation of the usability of the given digital tool for sexual health information. To achieve the research objectives, the following research questions are derived: (RQ1) What factors influence the acceptance of and behavioral intention of the named target group to use the digital tool for sexual health information? And second, (RQ2) how is the usability of the digital tool for sexual health information assessed by the mentioned target group?

## Methods

### Derivation of the used measurements

Technology Acceptance Models, like UTAUT, aim to explain effects of acceptability and intention to use of new technologies. The UTAUT model, developed by Venkatesh [18], unifies eight established research models including the Theory of Reasoned Action [19], Technology Acceptance Model (TAM) [20], Motivational Mode (MM) [21], Theory of Planned Behavior (TPB) [22], combined TAM and TPB (C-TAM-TPB) [23], Model of PC Utilization (MPCU) [24], Innovations-Diffusions-Theory (IDT) [25] and the Social Cognitive Theory (SCT) [26]. Nevertheless theUTAUT model outperformed all mentioned individual models [18].

The origin UTAUT model consists of four key constructs as exogenous variables, performance expectancy (PE), effort expectancy (EE), social influence (SI) and facilitating condition (FC), as well as the moderators age, gender, experience and voluntariness of use, which are described as direct determinants of the behavioral intention (BI) to use a system [18]. Venkatesh et al. considers the discriminance between the newly assembled constructs of the model. It shows that almost all of them are significantly correlated, but this correlation is in the low range. It can be concluded that the latent variables may influence each other, yet the constructs differ from each other [18]. The UTAUT model is applied in various studies, in context of new technologies and healthcare. The settings reach from health care service delivery [27], mobile health communication and/or information systems [7, 28, 29], acceptance and use of social media in the healthcare context [11] and mobile (sexual) health apps [16].

**Fig. 2:**
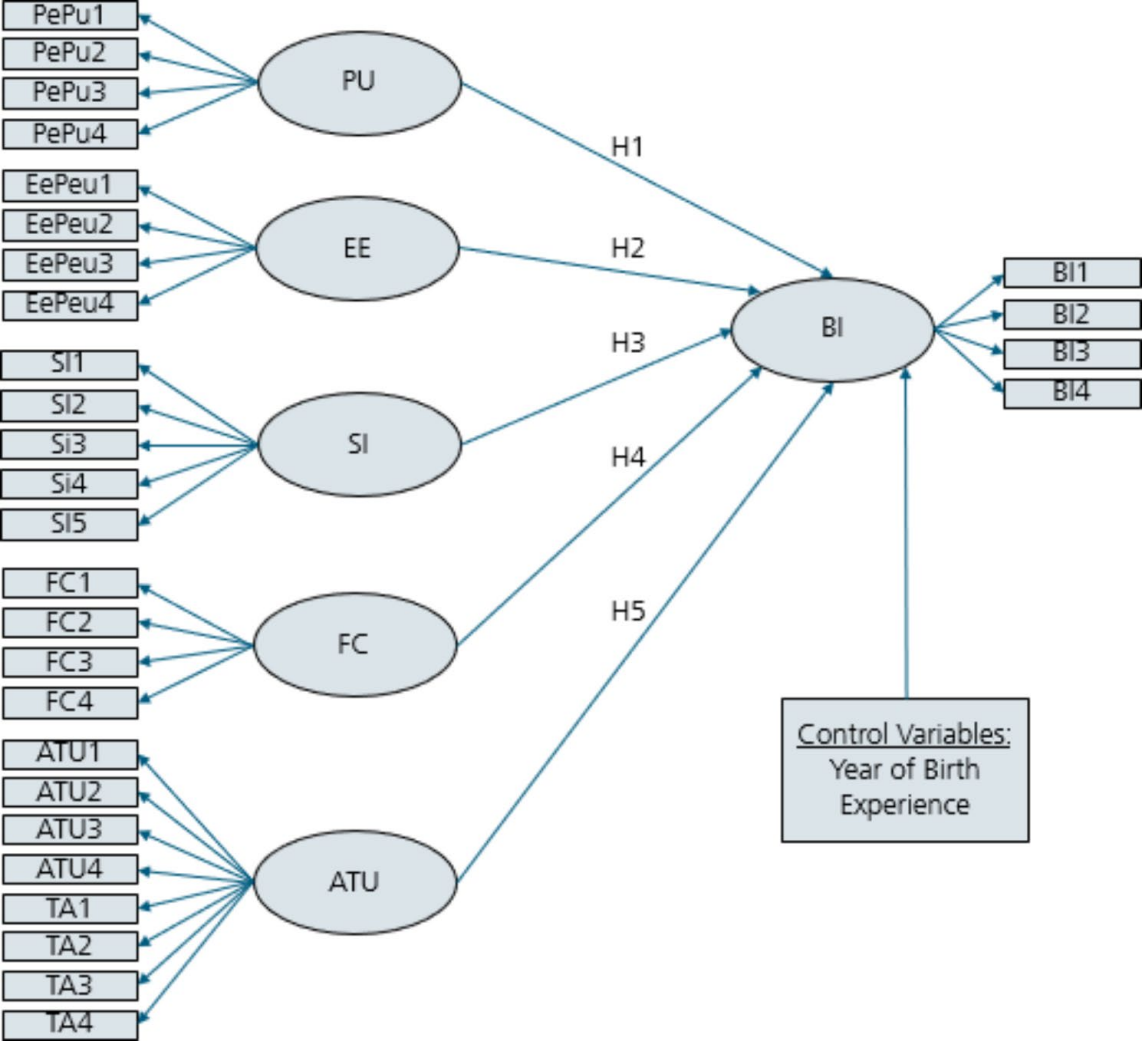
UTAUT: Research model of this study

To cover the particularities and complexity of healthcare setting respectively, several modifications have been proposed in the UTAUT model by the former mentioned studies. This is also the case in this study. Inspired by Olamijuwon et al. [11], the modified revision of the UTAUT model proposed by Dwivedi [30] is chosen as an appropriate theoretical framework for the study. The intention behind this, is to consider individual characteristics (users’ attitude towards technology and technological anxiety) that are not seen as direct determinants of the intention to use a system in the origin UTAUT model. In addition, the modified model is specified to the context of sexual health education for a digital tool in Kenya. Figure 2 shows the framework of the research model proposed to this study, based on UTAUT.

The constructs of the model, which may determine the intention to use a new technology (BI) and the corresponding hypotheses are described below.

**Performance expectancy** describes the factor of perceived usefulness (PU), which demonstrates the degree of a person’s belief that the usage of the new technology will help the user [18, 20]. Venkatesh et al. and Dwivedi et al. observed and underline the construct as a direct determinant influencing BI [18, 30]. Previous acceptance and usability studies, in the context of digital health, also indicate that the construct has a significant positive impact on BI [7, 11, 29]. Hence, the associated research hypothesis can be phrased as follows:

#### H1

PU has a significant impact on the behavioral intention to use the digital tool for sexuality information.

**Effort expectancy** (EE) describes the degree of perceived ease of use with the technology [18, 20], as this construct is determining to influence BI positively [18, 30]. In the context of the intention to use digital technologies in healthcare, some former studies, rejected a significant impact of EE on BI [8, 30–32]. However, an in-depth literature research shows that studies, explicitly, in context of sexual information and e-health technology in developing countries, were able to demonstrate a significant positive impact of EE on BI [11, 31]. Following this, the associated research hypothesis can be phrased as follows:

#### H2

EE has a significant impact on the behavioral intention to use the digital tool for sexuality information.

**Social influence** (SI) describes the perceived extent to which an individual believes that another person or group expects the individual user to use the technology. SI is seen as a direct determinant of BI [18]. The dynamic of the nature of cultural sensitive sexual health information, and the associated role of religion, are both considered, within the SI construct [11]. Previous studies mostly have found a significant positive impact of SI on the intention to use digital technologies in health-related topics [7, 11, 27, 29, 31], but also a negative impact was found in a related study [29]. The associated research hypothesis can be phrased as follows:

#### H3

SI has a significant impact on the behavioral intention to use the digital tool for sexuality information.

**Facilitating conditions** (FC) describes the perceived extent of an individual, supporting the existence of organizational and technical infrastructure to use of technology [18]. The construct is seen as a direct determinant of BI [30]. Previous research studies show a significant positive impact of FC on BI in the setting of mobile health communication and/or information systems [7, 28, 29, 31] as well as acceptance and use of social media in the healthcare context [11, 31]. The associated research hypothesis can be phrased as follows:

#### H4

FC has a significant impact on the behavioral intention to use the digital tool for sexuality information.

**Attitude towards technology use** (ATU) describes the positive and negative feelings of an individual about performing the targeted behavior, and is seen as a construct with a direct positive impact on BI [30]. Other context relevant studies also depict ATU as a construct with significant positive impact on BI [7, 11, 27]. Another study found a significant negative impact [7]. The associated research hypothesis can be phrased as follows:

#### H5

ATU has a significant impact on the behavioral intention to use the digital tool for sexuality information.

Supplemental to the UTAUT method, for a product-centered evaluation, the System Usability Scale (SUS) questionnaire is used. SUS was developed by John Brook in 1986 with the aim to create a simple standardized questionnaire to evaluate people’s perception of usability of a computer systems in a short time [32]. Since its creation, the questionnaire has been used in numerous scenarios and has become established as an effective, valid and reliable tool for assessing the usability of a wide variety of different products and services [33]. It functions as an industry standard and its widespread usage, makes it possible to compare the results, one has achieved with other products and systems [32]. In addition, a comparison of different versions of one product with itself, is also quick and inexpensive.

### Procedure and survey design

To explore the acceptability and intention to use of the developed digital tool for sexual health information (focusing on contraception product information and menstrual period) in Kenya a multimethod cross-sectional survey is used to collect information from a convenience sample. The total population of Kenya is 54.56 million in January 2023, of which 50.4% are female. Nearly 30% of the population is between 18 and 34 years old [34], which is roughly the targeted end-users of the developed digital tool, and addressed in this survey. As mentioned above, the theoretical basis of the study is mainly UTAUT (addressing RQ1) and complementary SUS (addressing RQ2). The ethic committee of the Witten/Herdecke University approved the research project on 06.08.2022 under the protocol code: S-119/2022.

For the procedure of quantitative research, all participants were offered a smartphone or a laptop, in case they do not have any digital device themselves. The volunteering participants had the opportunity to explore the developed prototype [17] for 20 min. They were able to read through all content and listen to the video files. After 20 min, the participants filled out the survey via smartphone or laptop.

The survey consists of three sections. Table 1 displays an overview of the questions. Section A contains a general information survey with nine questions to get demographic information (e.g., sex, age, education level, work status) and information about electronic device ownership and type of use regarding to health information from respondents. In section B, six questions are used to elicit responses to different statements, appropriate to the six constructs of our specified model. The statements serve as multiple questionnaire items, adopted from Venkatesh et al. and rewritten for the purpose of this study [18]. There are between four and eight statements per construct. All constructs are measured on a 7-point Likert scale from 1 (strongly disagree) to 7 (strongly agree).

**Table 1:** Questionnaire of the survey

**Section A**			
**Question English**			**Question Swahili**
My gender is ...			Jinsia yangu ni ...
Where are you located?			Mahali uliko?
What is your year of birth?			Mwaka wa kuzali?
What is your current working status?			Hali yako ya ajira?
What is your highest educational level?			Kiwango chako cha juu cha elimu?
What is your relationship status?			Hali ya Uhusiano wako ni gani?
Do you own any of the following items? Computer; Smartphone, Tablet, None			Je, unamiliki mojawapo ya hivi vifaa? Tarakilishi; Simu ya rununu ya ‘Android’; Simu ya Tabuleti; La
If you want to get specific information. How often do you ... Search online for information; Search online for health information; Search online for sexual health information			Ikiwa unataka kupata habari maalum. Ni mara ngapi wewe... kutafuta ujumbe kwa mtandao; tafuta ujumbe wa afya mtandaoni; kutafuta ujumbe wa afya ya kujumuiana kingono mtandaoni
Please rate your digital experience.			Je, matumizi yako ya kidijitali ni mazuri kwa kiasi gani? Tafadhali pima utajriba wako wa ubunifu wa kisasa.
**Section B - UTAUT**			
**Construct**	**Item**	**Question English**	**Question Swahili**
PU (perceived usefulness- performance expectancy)	PePu1	I find the given digital tool useful for providing sexual health information.	Naona kwamba kifaa hiki cha kidijitali ni cha muhimu kwa kusaidia kwa habari ya afya ya uzazi/kujumuiana.
	PePu2	Using the given digital tool would make it easier to inform myself and others about sexual and reproductive health.	Kutumia kifaa hiki cha kidijitali kunarahisisha mimi na wengine kujipatia habari (ujumbe) kuhusu afya ya kijinsia na uzazi.
	PePu3	Using the given digital tool would contribute to improvements in my sexual and reproductive health.	Kwa kutumia kifaa hiki cha kidijitali kunawezesha kuchangia kuboresha afya yangu ya kijinsia na uzazi.
	PePu4	Using the given digital tool for sexual health information would enhance myself to make better and more informed decisions about my sexual and reproductive health.	Kwa kutumia kifaa hiki cha kidijitali kwa ujumbe wa afya ya ngono kunaniwezesha kufanya uamuzi kuhusu afya yangu ya kijinsia na uzazi.
EE (effort expectancy- perceived ease of use)	EePeu1	Learning to operate the digital tool for sexual health information would be easy for me.	Kujifunza jinsi ya kutumia kifaa cha kidijitali kwa kupata habari (ujumbe) kuhusu afya ya kijinsia ni rahisi kwangu.
	EePeu2	I find the digital tool is easy to use.	Napata kifaa hiki cha kidijitali ni rahisi kutumia.
	EePeu3	My interaction with the digital tool to access sexual health information would be/is clear and understandable.	Kutangamana kwangu na kifaa hiki cha kidijitali ili kupata habari kuhusu afya ya kijinsia ni dhahiri na ya kueleweka vizuri.
	EePeu4	It would be easy for me to become skillful at using the digital tool for sexual health information.	Ni rahisi kwangu kuwa mjuzi wa kutumia kifaa cha kidijitali cha kupata habari (ujumbe) kuhusu afya ya kijinsia.
SI (social influence)	SI1	People who are important to me would disapprove of me using the digital tool for sexual health information.	Watu walio muhimu kwangu wanaweza kutokubaliana na mimi kutumia kifaa cha kidijitali cha kupata habari (ujumbe) kuhusu afya ya kijinsia.
	SI2	People who influence my behaviour would disapprove of me using the digital tool for sexual health information.	Watu wanao shawishi na kuathiri tabia yangu wanaweza kutokubaliana na mimi kutumia kifaa cha kidijitali kuhusu habari (ujumbe) wa afya ya kijinsia.
	SI3	My family would disapprove of me using the digital tool for sexual health information.	Familia yangu inaweza kutokubaliana na mimi kutumia kifaa cha kidijitali kuhusu habari (ujumbe) wa afya ya kijinsia.
	SI4	People who share the same religious belief will disapprove of me using the digital tool for sexual helath information.	Watu wenye Imani sawa na mimi wanaweza kutokubaliana na mimi kutumia kifaa cha kidijitali kuhusu habari (ujumbe) wa afya ya kijinsia.
	SI5	My religious belief does not support interacting with sexual health information on a digital tool even if it is made by a credible organization.	Imani yangu ya kidini yangu hainiruhusu kutangamana na habari (ujumbe) za afya ya kijinsia kwa kifaa cha kidijitali hata kama kimetengenezwa na shirika tajika.
FC (facilitating conditions)	FC1	I have the resources necessary to use the digital tool (e.g. wifi, laptop, smartphone).	Niko na raslimali muhimu za kutumia kwa kifaa cha kidijitali (kwa mfaano[k.m] uwezo wa mtandao, tarakilishi, simu ya mkono/rununu).
	FC2	I have the knowledge necessary to use the digital tool (e.g. basic digital knowledge).	Niko na maarifa ya kutumia kifaa cha digitali kama vile maarifa ya kimsingi ya kidigitali.
	FC3	Using the digital tool to get sexual health information fits well with the way I like to get information.	Kutumia kifaa cha kidijitali cha kupata habari (ujumbe) kuhusu afya ya kijinsia inaambatana na vile mimi hutaka kupata habari (ujumbe).
	FC4	I think that I would use the help of a technical person to use the digital tool for sexual health information.	Nadhani naweza kuhitaji huduma za mtaalam mwenye ujuzi aweze kunisaidia kutumia kifaa cha kidijitali kupata habari (ujumbe) kuhusu afya ya kijinsia.
ATU (attitude towards technology use)	ATU1	Using the digital tool for sexual health information is a good idea.	Kutumia kifaa cha kidijitali ili kupata habari (ujumbe) kuhusu afya ya kijinsia ni wazo zuri.
	ATU2	Accessing sexual health information through a digital tool is more comfortable than searching other internet sources like google.	Kupata habari (ujumbe) kuhusu afya ya kijinisia kupitia kifaa cha kidijitali ni bora zaidi kuliko kutumia mitandao mingine ya kutafuta habari kama google.
	ATU3	I would have fun using the digital tool for sexual health information.	Naweza kuwa na furaha kwa kutumia kifaa cha digitali kupata habari (ujumbe) kuhusu afya ya ngono.
	ATU4	Using the digital tool for sexual health information is interesting.	Kutumia kifaa cha kidijitali kupata habari (ujumbe) kuhusu afya ya kijinsia inavutia.
	TA1	Using the digital tool to seek sexual health information would make me very nervous.	Kutumia kifaa cha kidijitali kupata habari (ujumbe) kuhusu afya ya kijinsia yaweza kunitia wasiwasi.
	TA2	Using the digital tool to seek sexual health information may make me feel uncomfortable.	Kutumia kifaa cha kidijitali kupata habari (ujumbe) kuhusu afya ya kijinsia yaweza kunikozesha amani.
	TA3	Using the digital tool for sexual health information is a bad idea.	Kutumia kifaa cha kidijitali kupata habari (ujumbe) kuhusu afya ya kijinsia sio wazo zuri.
	TA4	Using the digital tool for sexual health information is unpleasant.	Kutumia kifaa cha kidijitali kupata habari (ujumbe) kuhusu afya ya kijinsia haipendezi.
BI (Behavioral Intention to use a digital tool for sexual health information)	BI2	I expect that I would use the digital tool in the future to search for sexual-health-related information.	Natarajia kutumia kifaa cha kidijitali siku zijazo kutafuta habari (ujumbe) unaoambatana na afya ya kijinsia.
	BI3	I would use the digital tool for information about contraceptive products.	Naweza kutumia kifaa cha kidijitali kutafuta habari (ujumbe) kuhusu mbinu za kupanga uzazi.
	BI4	I would use the digital tool for information about my menstrual period.	Naweza kutumia kifaa cha kidijitali kutafuta habari (ujumbe) kuhusu hedhi yangu.
	BI5	I plan to use a digital tool to get sexual health information.	Napanga kutumia kifaa cha kidijitali kupata habari (ujumbe) kuhusu afya ya kijinsia.
**Section C - SUS**			
**Question English**			**Question Swahili**
I think that I would like to use this system frequently.			Nadhani ningependa kutumia hii mbinu mara kwa mara.
I found the system unnecessarily complex.			Nilipata mbinu hii kuwa ngumu kiasi.
I thought the system was easy to use.			Nadhani mbinu hii ni rahisi kutumia.
I think that I would need the support of a technical person to be able to use this system.			Nadhani nitahitaji msaada wa mtaalam mwenye ujuzi ili niweze kutumia kifaa hiki.
I found the various functions in this system were well integrated.			Nilipata sehemu mbali mbali za mbinu hii zimeunganishwa vyema.
I thought there was too much inconsistency in this system.			Nafikiri kuna sehemu ambazo sio sahihi kwa mbinu hii.
I would imagine that most people would learn to use the system very quickly.			Nikidhani, watu wengi wanaweza kujifunza kutumia mbinu hii kwa wepesi.
I found the system very cumbersome to use.			Nilipata mbinu hii kuwa ya kuchosha kutumia.
I felt very confident using the system			Nalihisi kuwa na ujasiri kutumia mbinu hii.
I needed to learn a lot of things before I could get going with this system.			Nahitaji kujifunza mambo mengi zaidi kabla ya kuanza kutumia mbinu hii.

Section C contains the standardized SUS questionnaire. It is a 10-item-questionnaire measuring on a 5-point Likert response format scale from 1 (strongly disagree) to 5 (strongly agree).

Pre-testing was conducted, using a sample size of women presented in the community centers in Kenya, as well as women in Germany. The reporting of the e-survey results, the Checklist for Reporting Results of Internet E-surveys (CHERRIES) as suggested by Eysenbach, is considered, see Table 2.

**Table 2:** CHERRIES for used web survey

Item Category	Checklist Item	Explanation
Design	Describe survey design	The target population are primarily young women in Kenya. Therefore we placed the study in community centers, girl schools and universities in Kenya. The sample is not a convenience sample.
IRB (Institutional Review Board) approval and informed consent process	IRB approval	The ethic committee of the university of Witten/Herdecke approved the survey on 06.08.2022 under the protocol code: S-119/2022.
	Informed consent	Participants were informed on the welcome page of the survey. The Information and Consent for Participation in Research Study have been provided in English and Swahili. The participants in the study remain anonymous. By clicking the checkbox consent was confirmed.
	Data protection	The survey was hosted and all data were stored on its own secure server. No personal information was linked to survey results in any way.
Development and pre-testing	Development and testing	The used methods (UTAUT & SUS) consists of mainly standardised questions, which have been proved in various previous studies. Pre-testing was conducted using a sample of women in the community center in Kenya as well as women in Germany.
Recruitment process and description of the sample having access to the questionnaire	Open survey versus closed survey	The survey was an open survey.
	Contact mode	The study was placed in community centres, girl schools and universities in Kenya. This was supported by the social initiative of Boehringer Ingelheim, Making More Health, (MHH). The participants were able to share the link to the study with friends e.g. via WhatsApp, Instagram and Facebook.
	Advertising the survey	The study was announced by the contact persons in the community centres, girl schools and universities.
Survey administration	Web/E-mail	The survey was hosted on its own web server by the University Witten/Herdecke in Germany, using the software LimeSurvey.
	Context	The landing page of the survey was publicly accessible and distributed through an URL. This ensured that participants were able to share the survey.
	Mandatory/voluntary	The survey was completely voluntary. Users could access the landing page without completing the survey.
	Incentives	No incentives were offered to participants.
	Time/Date	The survey period was from 01.12.22 to 31.01.2023.
	Randomization of items or questionnaires	Survey items were not randomized.
	Adaptive questioning	No adaptive questioning was used.
	Number of Items	Section A: 9 questions Section B: 6 questions with 4–8 statements to be assessed Section C: 10 standardized questions Section D: 3 open-ended questions
	Number of screens (pages)	One welcome page and 9 pages with survey items
	Completeness check	Most survey items were mandatory, and respondents were prompted to complete outstanding items before leaving the survey page.
	Review step	Participants were able to review and change their answers by clicking the Back button.
Response rates	Unique site visitor	No cookies or IP controls were used to ensure that people could participate consecutively from the same device. Participation devices were brought to the survey.
	View rate (Ratio of unique survey visitors/unique site visitors)	Not measured.
	Participation rate (Ratio of unique visitors who agreed to participate/unique first survey page visitors)	Not measured.
	Completion rate (Ratio of users who finished the survey/users who agreed to participate)	Section A: 77/77 = 100% Section B: 65/77 = ~ 84% Section C: 64/77 = ~ 83% Section D: 63/77 = ~ 82%
Preventing multiple entries from the same individual	Cookies used	No Cookies were used.
	IP check	No cookies or IP controls were used.
	Log file analysis	Indicate whether other techniques to analyze the log file for identification of multiple entries were used. If so, please describe.
	Registration	Not necessary, since the survey was an open survey.
Analysis	Handling of incomplete questionnaires	Only completed questionnaires were included in the analysis.
	Questionnaires submitted with an atypical timestamp	Not used.
	Statistical correction	No statistical correction procedures or weightings were used in the analysis.

### Participants

The survey period was from 01.12.2022 to 31.01.2023. The online survey was available during the period and is distributed via contact persons in community centers, girl schools, and universities in rural areas in Kenya, using a convenience sampling technique. The target group of this study is predominantly female, from rural areas in Kenya between the age of 18–35 years. To support the participants and to achieve a higher response quote, the survey has been also accompanied in person from 11.01.2023 to 23.01.2023. For this purpose, various community centers in Eldoret, of Making More Health (MMH), as well as MOI University in Eldoret and Learning Lions have been visited for three days each. To create a pleasant atmosphere for the sensitive topic, the personal accompaniment consists of a woman from the Fraunhofer ISST in Germany and a trusted person from the respective institution on site. This procedure had the aim to counteract possible problems of comprehension in terms of content and language.

### Statistical analysis

The statistical program R [35] and in particular the package lavaan [36] were used for data analysis. Based on our multimethod approach, the data analysis is split into three parts: [1] a descriptive analysis [2], a SEM, and [3] an evaluation of SUS-model. First, a descriptive analysis is used to describe the sample characteristics of the 77 respondents. Second, the SEM, consisting of CFA and linear regression [37] Klicken oder tippen Sie hier, um Text einzugeben. is used to analyze the relationships in the UTAUT model and to test the hypotheses. Further, an evaluation of SUS is carried out according to Brooke [32]. For the five positively worded questions, the score contribution is the numerical scale position. Respectively, for the remaining five negatively worded questions the score contribution is the reverse scale position. The overall value of SUS amounts to a value between 0 and 100 [32]. As a rule of thumb, with a score of 68 or above, SUS is considered as good [32, 33].

## Results

### Descriptive analysis

A total of 96 people took part in the survey. About N = 77 responses were assessed as complete and consequently included in the analysis. The descriptive characteristics of the participants are shown in Table 3.

**Table 3:** Description Sample Characteristics

Item	Characteristics in Percentage (Frequency)				
Gender	**Female**	**Male**	**Diverse**		
	89.6% (69)	9.1% (7)	1.3% (1)		
Location	**Kenya**	**Germany**	**Another African Country**		
	93.5% (72)	3.9% (3)	2.6% (2)		
Age	**Below 20 Years**	**20–30 Years**	**Above 30**		
	16.9% (13)	68.9% (53)	14.3% (11)		
Working Status	**Student**	**Unemployed**	**Working**	**Others**	
	36.4% (28)	32.5% (25)	26% (20)	5.2% (4)	
Education	**Primary Education**	**Secondary Education**	**Post-Secondary Education**	**Tertiary Education**	**Prefer not to answer**
	13% (10)	35.1% (27)	14.3% (11)	36.4% (28)	1.3% (1)
Relationship Status	**Married**	**In a relationship, not married**	**Not in a relationship**	**Others or prefer not to answer**	
	32.5% (25)	29.9% (23)	27.3% (21)	10.4% (8)	
Digital Experience	**Excellent**	**Good**	**Average**	**Poor**	
	20.8% (16)	35.1% (27)	36.4% (28)	7.8% (6)	
Online search behavior for ... information	**Daily**	**Several days a week**	**About once a week**	**Less often**	**Never**
general	49.4% (38)	11.7% (9)	6.5% (5)	15.6% (12)	16.9% (13)
health	7.8% (6)	20.8% (16)	20.8% (16)	36.4% (28)	14.3% (11)
sexual health	3.9% (3)	19.5% (15)	16.9% (13)	44.2% (34)	15.6% (12)

As intended, the study main respondents are women, located in rural Kenya. Most participants preferred to fill out the survey in English, over their tribal dialect, as Swahili has many different forms, is constantly changing, and many are not able to understand it. Participants between 20 and 30 years constitute the largest group, of respondents, specifically, most of them were born in 2000 (17/77, 22%). In line with this observation, most participants report being a student, followed by a current working status as unemployed and working. Most of the participants have secondary or tertiary education. The respective relationship status is relatively evenly represented. In terms of owning technical items, it is noticed that, despite one person, every participant who owns technical devices has at least a smartphone. Regarding online search behavior it is shown, that the more specific the search intention towards sexual health, the less often an online search takes place. Most participants described their digital experience as, at least as good. Through this we assume that the participants can compare the given tool with other digital experiences and use their experiences to navigate through the tool. The items, working status, education, relationship status and digital experience will be examined in context to the latent construct of BI, more precisely later. As the items are aiming to be included in the selection for the following regression analysis.

Table 4 displays mean and standard deviation per item of the questionnaire. A 7-point scale was applied to all questions. With a mean value resulting in of 5.9 to 6.1, the answers to the eight questions of the first both constructs, perceived usefulness, and perceived ease of use, are close to each other and indicate good feedback on the digital tool. The four questions of the BI construct, also perform well. The averages are close to each other. The mean values of the questions on SI are in the lower half of the scale. The scatter is larger than for the previously mentioned constructs. The moments on facilitating conditions do not give a consistent pattern. At ATU, the answers to the first four questions are in the upper range of the scale. The negatively directed questions’ averages TA2 - TA4 are in the lower range. TA1 stands out with a comparatively high mean value of 4.0. However, the standard deviation is also relatively high.

**Table 4:** Descriptive Statistics of Item Responses

Item	Mean	Standard Deviation
**Performance Expectancy - Perceived usefulness (PU)**		
PePu1	5.8	1.6
PePu2	5.9	1.5
PePu3	6.1	1.2
PePu4	5.9	1.3
**Effort Expectancy - Perceived ease of use (EE)**		
EePeu1	6.1	1.1
EePeu2	6.0	1.1
EePeu3	6.1	0.8
EePeu4	6.0	1.3
**Social Influence (SI)**		
SI1	3.0	2.1
SI2	3.0	2.1
SI3	2.6	1.8
SI4	2.8	1.8
SI5	2.7	1.9
**Facilitating Conditions (FC)**		
FC1	4.9	2.2
FC2	6.0	1.3
FC3	5.7	1.5
FC4	4.4	2.3
**Attitude towards technology use (ATU)**		
ATU1	6.3	1.0
ATU2	6.0	1.5
ATU3	5.9	1.5
ATU4	6.2	1.1
TA1	4.0	2.1
TA2	2.5	1.6
TA3	2.0	1.3
TA4	2.0	1.6
**Behavioral intention to use the digital tool for sexual health information (BI)**		
BI1	6.1	0.9
BI2	6.2	1.1
BI3	6.2	1.2
BI4	6.2	1.0

### Structural equation model

It was decided to use SEM in this case, which is a CFA, followed by regression to address the evaluation of the questionnaire, as a measuring instrument for the latent variables. Χ^2^ test for model conformity, performing on the null hypothesis, that the predicted model and observed data are equal. Further Comparative Fit Index (CFI), Root Mean Square Error of Approximation (RMSEA) and Standardized Root Mean Residual (SRMR) were considered.

First, the model was specified, according to the UTAUT model (see Fig. 2). To identify the model, the variances of the latent variables in the CFA were set to 1. 12 observations were removed, due to missing values. Hence the number of observations used was 65 (65/77, 84.4%).

Considering factor loadings and internal correlations, it was noted that questions FC1 - FC4 (s. Table 1), which correspond to the FC construct, do not tend to indicate a common latent variable. Moreover, the standardized factor loading of FC3, leads to a numerical problem: the factor loading is estimated to be > 1 and the resulting estimated variance, is negative. Additionally, this also clearly indicates a content error in the model. Therefore, the items FC1 - FC4 are removed from the model.

**Fig. 3:**
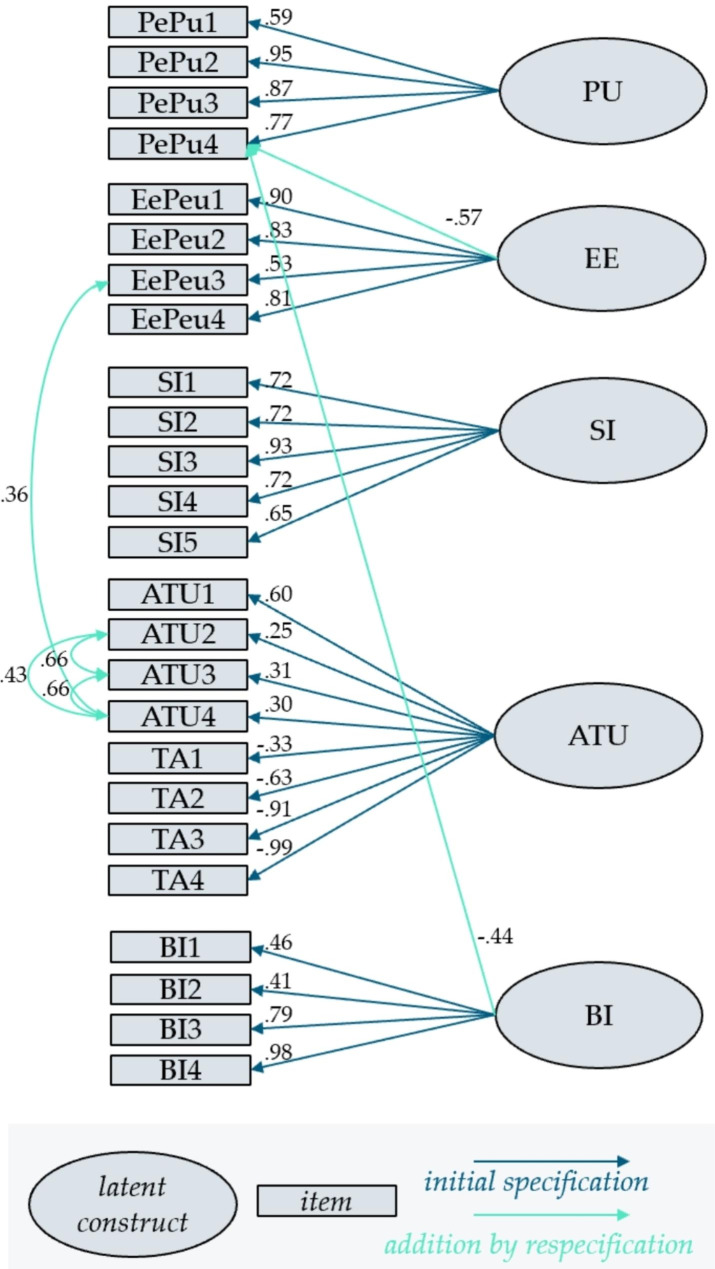
Final respecified factor model

The next step intends to improve the indices by extending the model, and thus arrive at a better-fitting model. For this purpose, the correlation between items, as well as the use of one item for several latent variables, will be allowed. Hence, the modification indices are computed. The proposals for respecification are selected step by step. For each decision, question whether the extension is meaningful, in terms of content, and select the variant that promises a large improvement in the Χ^2,^ distribution, test statistic for the hypothesis of model conformity. The use of the items BI1- BI4, within the other constructs, is avoided. As this is supposed to be our target variable. After several respecification steps, the model shown in Fig. 3 is obtained. The corresponding CFA output, concerning factor loadings and interactions, are shown in Table 5.

**Table 5:** CFA output concerning factor loadings and interactions

Latent Variable	Item	Estimate	Std. Error	z-value	P(>|z|)	Std.all
PU	PePu1	0.86	0.17	5.13	< 0.001	0.59
	PePu2	1.35	0.14	9.98	< 0.001	0.95
	PePu3	1.06	0.12	8.73	< 0.001	0.87
	PePu4	1.00	0.14	6.88	< 0.001	0.77
EE	EePeu1	0.97	0.11	8.96	< 0.001	0.90
	EePeu2	0.88	0.11	7.89	< 0.001	0.83
	EePeu3	0.43	0.09	4.90	< 0.001	0.53
	EePeu4	1.00	0.13	7.61	< 0.001	0.81
	PePu4	0.74	0.12	6.28	< 0.001	0.57
SI	SI1	1.49	0.23	6.50	< 0.001	0.72
	SI2	1.48	0.23	6.52	< 0.001	0.72
	SI3	1.66	0.18	9.43	< 0.001	0.93
	SI4	1.32	0.20	6.50	< 0.001	0.72
	SI5	1.22	0.22	5.67	< 0.001	0.65
ATU	ATU1	0.57	0.11	5.26	< 0.001	0.60
	ATU2	0.38	0.18	2.06	0.04	0.25
	ATU3	0.45	0.18	2.51	0.01	0.31
	ATU4	0.31	0.12	2.58	0.01	0.30
	TA1	-0.70	0.26	-2.70	0.01	− 0.33
	TA2	-1.03	0.18	-5.65	< 0.001	− 0.63
	TA3	-1.20	0.13	-9.39	< 0.001	− 0.91
	TA4	-1.55	0.14	-10.93	< 0.001	− 0.99
BI	BI1	0.41	0.11	3.86	< 0.001	0.46
	BI2	0.46	0.14	3.42	< 0.001	0.41
	BI3	0.91	0.12	7.38	< 0.001	0.79
	BI4	1.01	0.10	10.05	< 0.001	0.98
	PePu4	-0.57	0.13	-4.31	< 0.001	− 0.44
**Interactions**						
**Item1**	**Item2**	**Estimate**	**Std. Error**	**z-value**	**P(>|z|)**	**Std.all**
ATU3	ATU4	0.94	0.20	4.61	< 0.001	0.66
ATU2	ATU3	1.35	0.30	4.44	< 0.001	0.66
ATU2	ATU4	0.63	0.19	3.37	< 0.001	0.43
EePeu3	ATU4	0.25	0.07	3.39	< 0.001	0.36

The Χ^2^ test statistic amounts to χ^2^_259_ *=* 532, *p* < .001 and is thus, compared to the original model, is still highly significant. The CFI improves remarkably by 0.16 to 0.75, compared to the primary model. An improvement can also be seen in the other indices, leading to RMSEA = 0.13 and SRMR = 0.14. However, the criteria of fit indices according to Hu and Bentler [38], is still not fulfilled.

The quality of the measurement model is evaluated by considering indicator reliability, internal reliability of the constructs, discriminant validity, as well as convergence validity.

Indicator reliability is measured through standardized indicator loadings. Figure 3 shows that for PU and EE, three out of four items are > |0.7|. The remaining are still satisfactory with > |0.5|. For EE, as mentioned, the item PePu4 is added, which has an acceptable charge. Four of five SI loadings SI are > |0.7|, one is only slightly below. For ATU, half of the loadings are < |0.4|, namely ATU2, ATU3, ATU4 and TA1. These items have negligible contribution to the measurement of the latent variable. With around |0.6| the loadings of ATU1 and TA2 can be classified as moderate. Finally, considering the factor loadings of BI. BI1 and BI2 load rather weakly on the latent variable, with values between |0.4| and |0.5|. BI3 and BI4 on the other hand are remarkably strong. Again, PePu4 has a moderate loading on BI, as an addition respecification.

Internal reliability is examined, considering Cronbach α, as shown in Table 6. It turns out that PU, EE as well as SI, show a very good strength of association and the indicator for BI is classified as good, according to the rules of thumb [39]. ATU, on the other hand, is somewhat weak. One should keep in mind, that with eight questions, remarkably more items are chosen for this construct, compared to others.

**Table 6:** Cronbach α

PU	EE	SI	ATU	BI
0.84	0.83	0.86	0.43	0.70

Convergent validity and discriminant validity are assessed by considering the correlation between the latent variables of Table 7.

**Table 7:** Correlation Between Latent Variables

	EE	SI	ATU	BI
PU	0.10	0.11	0.20	0.50
EE		− 0.21	0.16	0.23
SI			− 0.46	0.03
ATU				0.32

The discrimination between SI and ATU is rather unsatisfying. On one hand, a greater convergence between BI, and on the other latent variables would be desirable. The highest of these correlations is between BI and PU.

The second step of SEM focuses on the question, of what influence the constructs and variables have on the latent target variable BI.

**Fig. 4:**
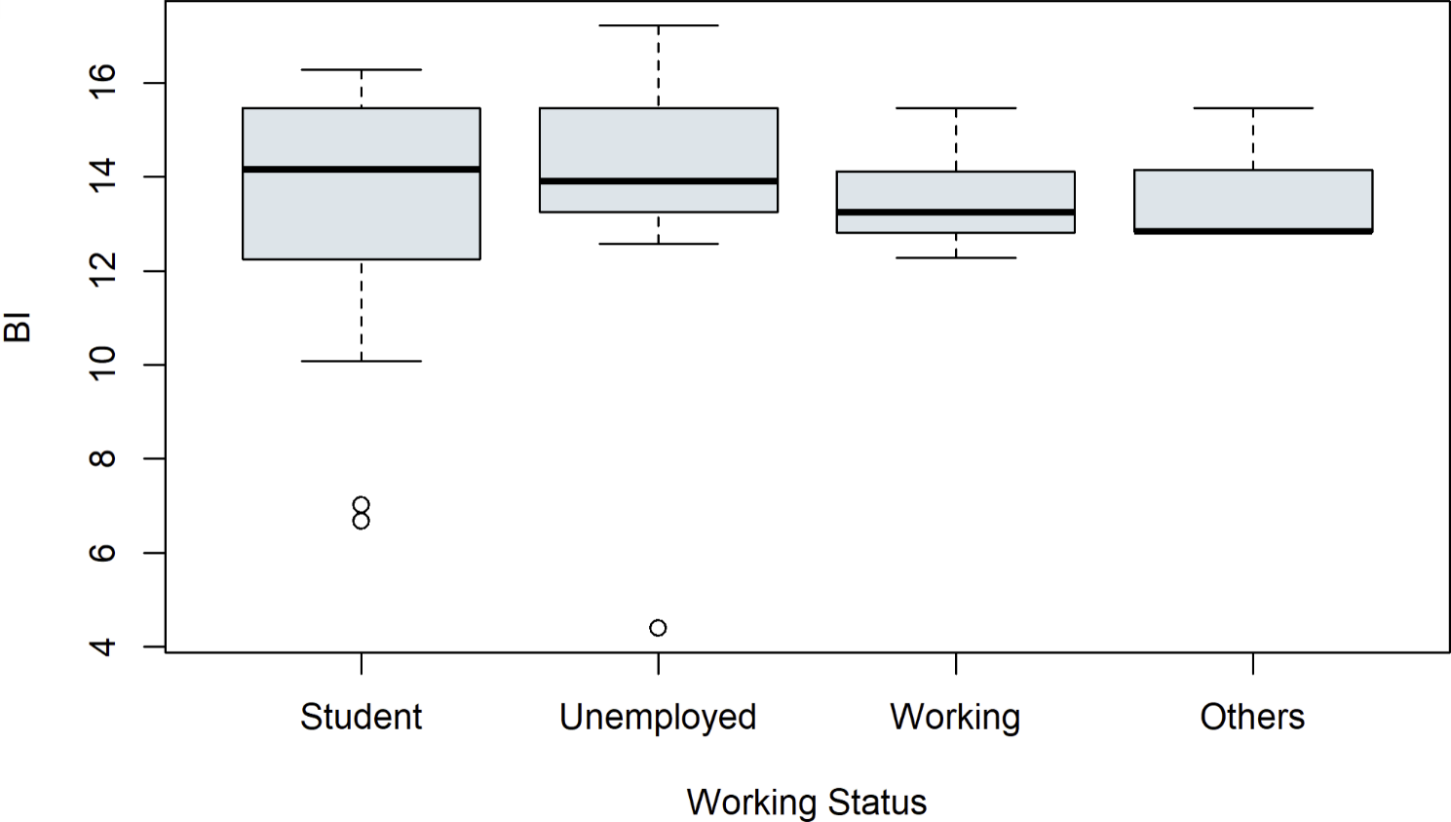
Boxplots of the latent target variable BI depending on the working status of the study participants

**Fig. 5:**
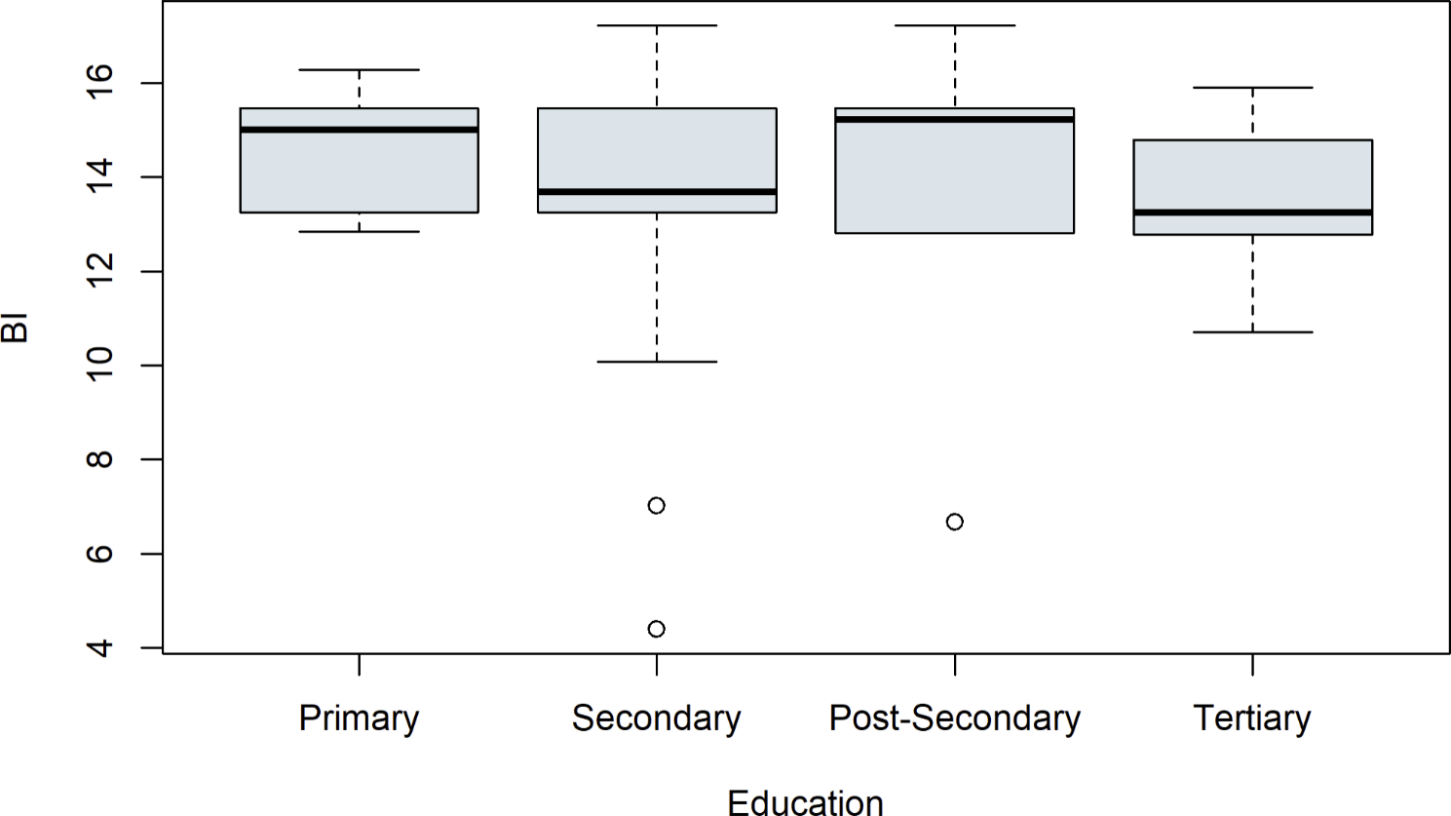
Boxplots of the latent target variable BI depending on the education of the study participants

**Fig. 6:**
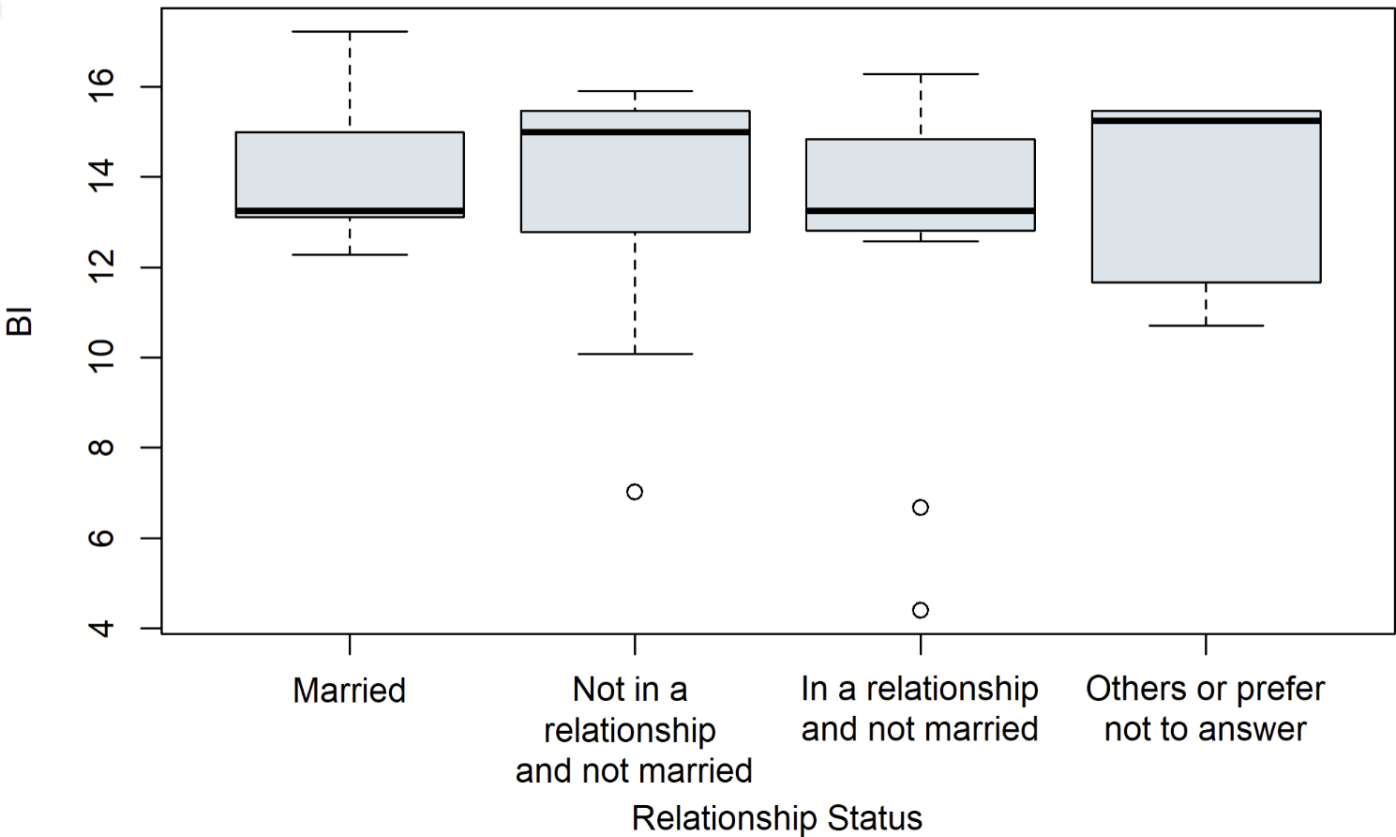
Boxplots of the latent target variable BI depending on the relationship status of the study participants

**Fig. 7:**
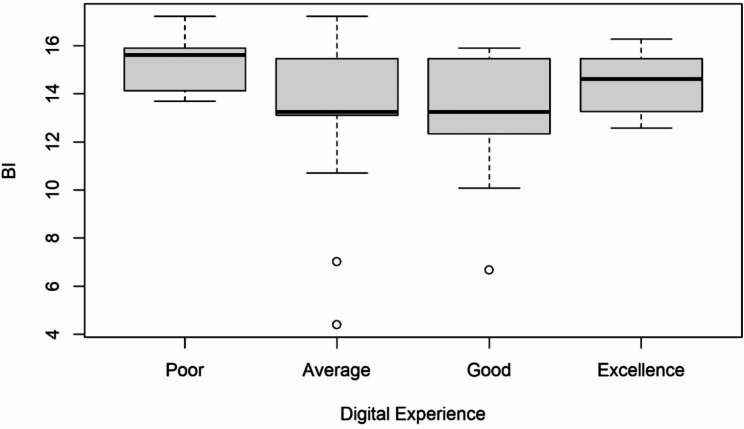
Boxplots of the latent target variable BI depending on the study participants’ digital experience with the tool

First, some control variables were considered. Boxplots were used to examine whether the attributes of the categorial variables show a difference on the outcome of the latent construct BI. See Fig. 4 for the control variable working status, Fig. 5 for education, Fig. 6 for relationship status and for digital experience see Fig. 7. No evident structural variations can be found, because the boxes overlap to a large extent. Therefore, these items were not used for further investigation.

**Fig. 8:**
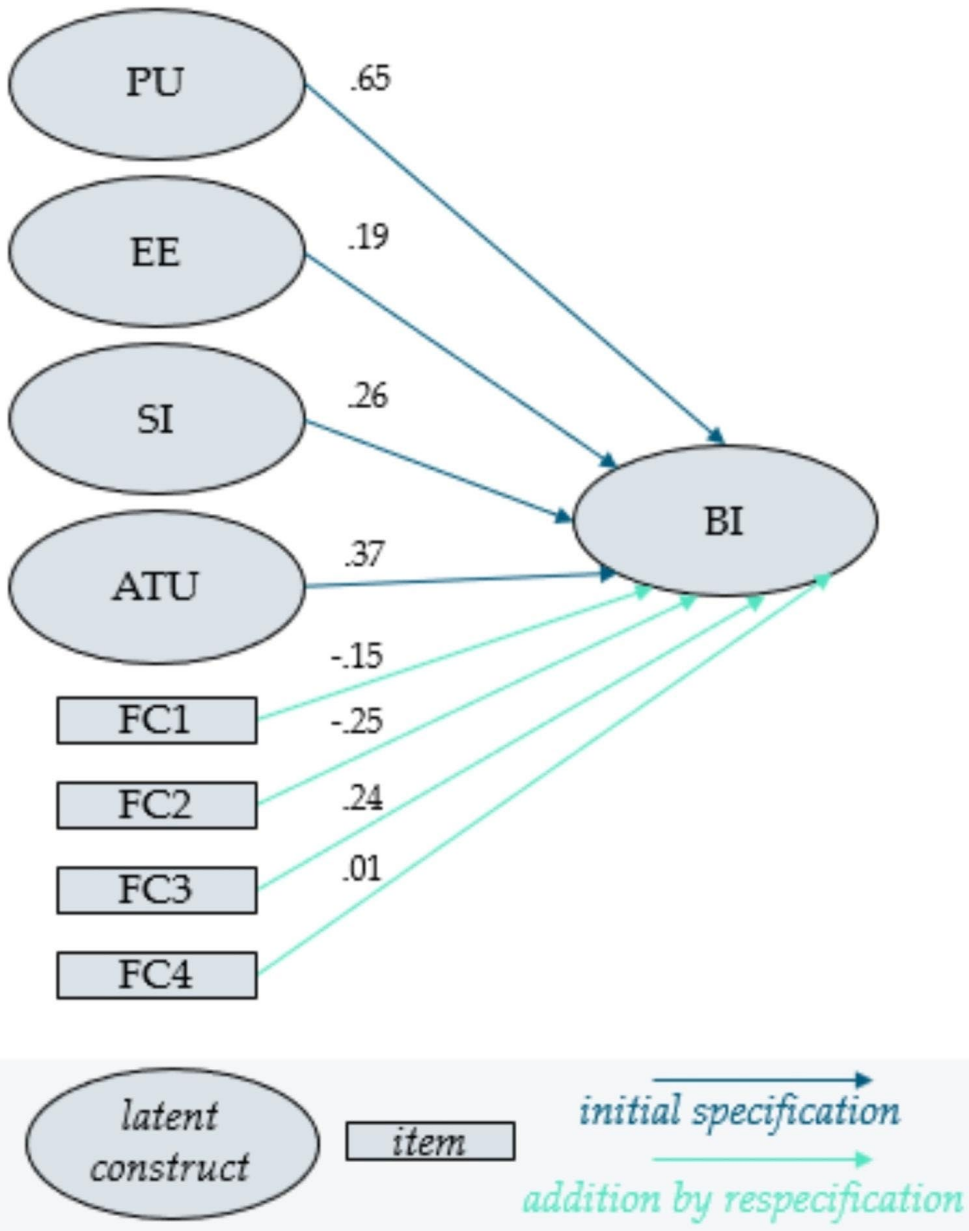
Regression Model: Architectural illustration

Subsequent to the factor analysis, a multiple linear regression is employed to identify relationships between the eight identified independent variables (PU, EE, ATU, SI, FC1, FC2, FC3, FC4) and one dependent variable (BI). The results, depicted in Table 8; Fig. 8, show that the constructs PU, EE, SI and ATU, as well as FC3, positively influence the behavioral intention to use the digital tool for sexual health information, while FC1 and FC2 influence BI negatively. The usefulness of the tool (PU) has a particularly strong influence on BI. Likewise, ATU, FC1, FC2 and FC3 have, by a level of 5%, a significant impact on the target. Item FC4, which is intended to collect information about whether the user would accept technical assistance from a person, has virtually no impact on the outcome of BI. Given the high variance of responses for item FC4 (M = 4.4, SD = 2.3), see Table 4 above, it cannot be due to the fact that technical assistance was not considered by the participants.

**Table 8:** Output regarding regression parameters

Predictors	Estimated Parameter	95% CI	Std. Err	z-value	*P* (>|z|)
PU	0.65	[0.31, 0.99]	0.17	3.75	< 0.001
EE	0.19	[-0.11, 0.49]	0.15	1.28	0.20
SI	0.26	[-0.08, 0.60]	0.17	1.55	0.12
ATU	0.37	[0.03, 0.71]	0.17	2.21	0.03
FC1	-0.15	[-0.27, -0.03]	0.06	-2.29	0.02
FC2	-0.25	[-0.47, -0.03]	0.11	-2.19	0.03
FC3	0.24	[0.04, 0.44]	0.10	2.39	0.02
FC4	0.01	[-0.11, 0.13]	0.06	0.24	0.81

R² is for the overall model 0.51, which implies that half of the variance for the dependent construct BI, is explained through the eight independent variables in the regression model.

The correlation between year of birth and BI amounts to − 0.11, which is quite weak. Adding year of birth as numerical predictors, does not improve the regression model and is therefore discarded.

In response to RQ1, based on the results of the multiple linear regression analysis the following hypothesis are supported or not evidenced:

#### H1

Perceived usefulness has a significant impact on the behavioral intention to use the digital tool for sexual information. à Supported.

#### H2

Perceived ease of use has a significant impact on the behavioral intention to use the digital tool for sexual health information. à Not evidenced.

#### H3

Social Influence has a significant impact on the behavioral intention to use the digital tool for sexual health information. à Not evidenced.

#### H4

Facilitating conditions have a significant impact on the behavioral intention to use the digital tool for sexual health information. à Differentiated considerations necessary.

#### H5

Attitude to use has a significant impact on the behavioral intention to use the digital tool for sexual health information. à Supported.

### SUS

There are 13 observations removed due to missing values. Hence the number of observations used for the SUS is 64 (64/77, 83.1%). Ten questions pertain to SUS, see Section C of the Questionnaire in Table 1, (s. Table 1) each with a 5-point scale. According to Brook [32], for positively worded questions (question 1, 3, 5, 7, 9), the score contribution is the scale position. Consequently, for negatively worded questions (question 2, 4, 6, 8, 10), the score contribution is the reversed scale position. To interpret the SUS score, it can be plotted on a scale from 0 to 100, with 100 being the best possible score. A SUS score of 67.3 is determined. The rating of the scale position can be done via acceptability scores or adjective ratings. Regarding acceptability, a score below 50 is described as unacceptable, above 70 as acceptable and in between as marginal. In terms of adjective ratings, from a score of 52, the SUS is considered as “okay” and from above average at 71 as “good” [32, 33]. Thus, the SUS score of this study can be classified as “high marginal” to nearly “acceptable” and in terms of adjective ratings described as “okay”, nearly “good”.

The RQ2, “How is the usability of the digital tool for sexual health information assessed?”, can therefore be answered with: The usability of the digital tool for sexual health information is assessed as ok and high marginal. This implies that there is still potential for improvement.

## Discussion

This study investigated the acceptance to use and usability of a newly developed digital tool for sexual health education of young adults in Kenya, concerning the utilization of a modified UTAUT model. The analysis and a comparison of the results with prior studies confirm the applicability of the modified revision of UTAUT model proposed by Dwivedi with additional specifications to meet the context of sexual health education throughout the given digital tool in Kenya.

Among the significant explanatory features, perceived usefulness has the greatest influence on the behavioral intention. It has also been depicted as a construct with significant effect on the intention to use, in prior studies [7, 18, 29, 30]. Specifically, aligning with former studies, it has been identified as the construct with the strongest impact on BI, compared to the other constructs [7, 18, 29]. Hence, the usefulness of the tool is in the first place for usage behavior. Participating women consider the tool to be useful for providing health information, which could lead to a contribution of their sexual and reproductive health improvement, as well as enhancing users to make better and more informed decisions for their mental and physical health state.

To consider the individual characteristics of the respondents (users’ attitude towards technology and technology anxiety), the origin UTAUT model of Venkatesh is extended by the construct ATU [11, 30]. The significant effects of the construct attitude to use on BI, conforms with results of Chilliers, Olamijuwon, and Dwiwedi [7, 11, 30]. The results indicate the positive and negative feelings of the women using digital tools for sexual health information, which influences the actual use. In addition, a rather weak internal consistency of ATU was observed. Therefore, for further research it is proposed to reduce the number of items, as the questions ATU2, ATU3, ATU4 and TA1 are less suitable for measuring the latent variable. It may be helpful to combine ATU2, ATU3 and ATU4 to one item, as they turn out to be highly correlated. Furthermore, one could consider reformulating the questions to improve the discrimination between the constructs ATU and SI.

Supplementary research could examine construct ATU more specifically. Potential research directions could include the investigation of the interactions between ATU and the other constructs. As well as examining the attitudes of young women in Sub-Sharan Africa towards digital sexual health information through an application what kind of attitude the participants have towards the digital tool for sexual health information tools. As well as how deep religious and cultural believes determine the level of education. Further the acceptability and intention to use a digital tool by men, to inform themselves about female sexual health, could be explored. This could contribute to a holistic sexual education approach, strengthen an understanding of female well-being, and create sensitivity towards sexual health as well as takeing over responsibility to make choices without religion and cultural stigmata.

Regarding the construct, facilitating conditions, a differentiated consideration of the results is necessary. This can be explained with the finding, of the not well represented latent construct by the underlying questions. The questions are ambiguous and represent several aspects. This can be explained by the heterogeneous nature of the questions within this construct. Therefore, the construct is not considered in the CFA, but the individual questions are included in the further analysis. Upcoming research could construct FC in a more differentiated way and divide it into several constructs.

Our results show that three out of four measured FC-items, have a significant impact on BI. Prior similar studies have also identified a significant impact of FC on BI [11, 28–30, 37, 40]. However, in contrast to the former mentioned studies, negative effects are evident. Based on the content of the question, one possible explanation for the negative effect of FC1 and FC2 could be, that people who have the necessary digital resources for getting informed available (smartphone, Wi-Fi, etc.), have the necessary knowledge to use a digital tool (e.g., basic digital knowledge). They do not have a high need for the developed web-based application, as they can get information from other digital sources. However, no direct effect between the degree of education and the intention to use the tool was apparent, as shown in Fig. 5.

The not evidenced statistically significant impact of effort expectancy on the participants BI, aligns with the results of Akinnuwesi, Chilliers and Khatun [7, 28, 29].

Regarding the significant impact of social influence, the literature shows opposing results. In contrast to some earlier studies [29], in this study, social influence has no significant impact on BI. In terms of acceptance and the use of a new cloud-based mHealth technology, Khatuns results also show, that SI has no significant effect on BI [28]. To interpret the results, it is necessary to consider the context and cultural surrounding of the participants in this study. There might be a selection bias, as people who were willing to participate in such a study in the first place do not mind talking about sexual health information and may tend to be more open-minded and progressive. This suggests that participants may feel less constrained by factors like family, friends, people with the same religious beliefs or religious belief itself, when using the given tool for sexual health information. The findings show a correlation between SI and the construct ATU, which indicates that SI and ATU influence each other, which is consistent with literature [11, 30], and with the observation during the procedure of the questionnaire. It was observed that the participants in rural regions of Kenya where willing and interested in using the tool, but perceived with a potential bias.

The fit indices CFI, SRMR, and RMSEA, as well as Cronbach α indicate a rough direction, but they should be viewed with caution for small data sets, as is the case here. Some rules of thumb may not be applicable.

Regarding RQ2, (How is the usability of the digital tool for sexual health information assessed by the mentioned target group?), the usability of the developed web-based application for sexual health information can be, with a SUS score of 67.3, interpreted as okay and high marginal. In context of digital tools for female sexual health, literature shows, SUS scores which are above a score of 70, for average usability [41, 42]. For example, Dubinskaya et al. investigated existing applications addressing female sexual health, focusing on the educational content and indicating for one application, a SUS score of 70, and for five further apps, a score of 97.5 [41]. In addition, SUS is also applied in terms of gamified applications digital interventions focusing on sexual health education. Here, literature reveals for a sexual health education mobile game a SUS score of 77 [15] and a score of 68.4 for a digital infertility prevention training [43]. When comparing the SUS scores of those studies with those in literature, it should be noted that the digital tool of this study is a prototype. Therefore, the obtained SUS score helps to rank the usability and identifies a potential for usability improvement. Further studies should examine the comparison between the prior mentioned digital tools and a SMS based sexual health information platform.

During the study, additional content was requested by the participants, after viewing the prototype, concerning cervical and breast cancer, as well as a parenting style and guidelines for young parents. The parenting and reproductive health knowledge shall be illustrated, easy and comprehendible, preferred in illustrated video format in English.

Nevertheless, there are limitations within the study that need to be taken into consideration. It should be considered that the generalization of results of the study regarding digital tools for sexual health information is limited to the specifically developed web-based application prototype, as well as too the defined constructs in Chap. 2.1, of the UTAUT model. In addition, a generalization should be tempered with caution, because a convenience sample is used and therefore findings may not be generalizable to a larger population. Throughout the use of SUS, a frequently used measurement, the lack of generalization is addressed, as well as a standard outcome to compare with other studies. Further research should consider, standardized measurements, like SUS, in addition to specified methods to the study context, in order to create comparability [44].

Based on the findings of this study, theoretical and practical implications can be derived. Starting with the theoretical implications, the results of the study contribute to theory in several ways.

Firstly, the research makes a fundamental contribution to theory, by enriching e-health research in the context of a very sensitive topic in a developing country and rural areas. By doing so, the results provide insights of theoretical of predictors with a significant impact on the behavioral intention to use e-health technology in this case, a digital tool for sexuality information by young adults, primarily women, between 18 and 35 in developing countries. Secondly, the study concludes, the modified revision of the UTAUT model, proposed by Dwivedi [30] and SUS, are applicable to digital health research and accordingly extend the theoretical application of the validated methods, to the intention to use a digital tool for sexual health education by young adults in Kenya. In addition, by using these methods the comparison basis in digital health research is extended. Nevertheless, it should be considered that the origin UTAUT model was not developed for the context of e-health research, and therefore adaptations to the own study context are advisable. Following this, inspired by Olamijuwon et al. [11], the origin UTAUT model was extended according to Dwivedi with determinants considering the individual characteristics, and further specify the modified model to the context of sexual health education through cultural norms in Kenya.

Derived from these studies, there is also a practical implication for the adoption of digital tools in context of sexual health education, in developing countries. The study findings provide practical information for the design and implementation of a digital health tool delivering sexual health information in a developing country, besides Soehnchen et al. [17]. Especially for e-health technology designers and researchers, the findings are beneficial to understand challenges of a specific context. As discussed before, considering the theoretical constructs PE, FC and ATU is imperative for the acceptance and intention to use a digital tool for sexual health education by young Kenyan adults.

## Conclusions

This research study describes a validated approach assessing acceptance and usability of a newly developed digital tool designed for sexual health education in Kenya. The study adopted the Unified Theory of Acceptance and Use of Technology (UTAUT) and the System Usability Scale (SUS) to this specific context, recognizing the need to account for unique cultural, religious, and educational factors in Kenya. The study places a distinctive focus on the acceptability and intention to use of the developed digital tool, which covers sensitive topics, such as contraception information, menstrual period education, and video educative materials concerning female genital mutilation. In doing so, the study hints the profound impact of the correlation on expectancy, social influence, facilitating conditions, behavior, and overall technology affiliation while shedding light on the specific context of local culture, religion, and educational levels on technology acceptance.

The findings reveal a high intention to use the digital tool, among participants, scoring approximately 6.1 to 6.2 on a 7-point scale to use. Highlighting that the newly developed digital tool, effectively fulfills its purpose of delivering comprehensive sexual health education.

Perceived expectancy (PU), and attitude to use (ATU) underline the verified positive impact on an individual user in its behavioral intention (BI) to use the digital tool for sexuality information, and show its potential, while significantly impacting the social influence (SI) factor. Social influence (SI) did not demonstrate statistical significance in this study, nevertheless, its presence should not be underestimated. Notably, participants situated in the rural community center, where the prototype was tested, exhibited a higher level of receptiveness to innovative tools, due to their pre-existing access to digital infrastructure. However, factors of facilitating conditions (FC) and effort expectancy (EE) differ in significance of impact in behavioral intention to use the digital tool. This specific context adds to the novelty of findings.

In conclusion, the study tailored survey methods and approach for acceptance and usability for sexual health education in Kenya, as well as the high intention to use, scores the tool’s potential effectiveness in addressing crucial sexual health topics, in this specific cultural and educational setting. Additionally, it paves the way for further investigation into the influence of cultural and religious factors on technology and digital educational tools.

## Data Availability

According to the guidelines for good scientific practice and research data management of Witten/Herdecke University, data can be requested by contacting the corresponding author.
